# Thiol-Functionalized Succinoglycan via Cysteine Grafting: Enhanced Rheological Properties and Antioxidant Activity

**DOI:** 10.3390/polym18070849

**Published:** 2026-03-31

**Authors:** Sobin Jeon, Kyungho Kim, Eunkyung Oh, Haemin Jin, Seunho Jung

**Affiliations:** 1 Department of Bioscience and Biotechnology, Microbial Carbohydrate Resource Bank (MCRB), Konkuk University, 120 Neungdong-ro, Gwangjin-gu, Seoul 05029, Republic of Korea; wjsthqls030414@naver.com (S.J.); rudgh971225@naver.com (K.K.); eunkyung_5@naver.com (E.O.); 2 Department of System Biotechnology, Microbial Carbohydrate Resource Bank (MCRB), Konkuk University, 120 Neungdong-ro, Gwangjin-gu, Seoul 05029, Republic of Korea; hahahaemin0101@gmail.com

**Keywords:** succinoglycan, cysteine modification, thiol-functionalized polysaccharide, rheology, antioxidant activity

## Abstract

Cysteine-modified succinoglycan (SG-Cys) was synthesized via EDC/NHS-mediated amidation by grafting cysteine onto succinoglycan isolated from Sinorhizobium meliloti. The successful introduction of cysteine moieties was confirmed by 1H NMR and FTIR analyses, while the degree of substitution was quantitatively determined using Ellman’s assay. The incorporation of cysteine significantly influenced the physicochemical and rheological properties of the polymer. In particular, SG-Cys exhibited up to a 1.8-fold increase in viscosity compared with native succinoglycan. The viscoelastic behavior of SG-Cys was systematically evaluated under various environmental conditions, including different pH, ionic strengths, temperatures, and polymer concentrations, revealing enhanced responsiveness to external stimuli. Radical scavenging assays demonstrated that SG-Cys displayed up to a 2.5-fold increase in antioxidant capacity compared with unmodified SG, as determined by DPPH and ABTS assays. Cytotoxicity evaluation using HEK-293 cells confirmed that the modified polymer exhibited no significant cytotoxic effects. Overall, the results demonstrate that thiol functionalization of succinoglycan effectively improves both rheological performance and antioxidant activity, suggesting that SG-Cys is a promising multifunctional bioactive polymer for potential applications in biomaterials, drug delivery, and bioengineering systems.

## 1. Introduction

Amino acids are fundamental building blocks of proteins and have been extensively investigated across various fields of biotechnology [1,2]. In recent years, the incorporation of amino acids into biomaterials has emerged as an effective strategy to enhance biocompatibility and introduce functional properties. Accordingly, increasing attention has been directed toward the utilization of amino acids in polymer platforms to engineer bioactive and functional materials [3,4]. Among them, cysteine, a naturally occurring amino acid containing a thiol group (–SH), has attracted particular interest due to its high chemical reactivity and inherent biocompatibility [5,6].

The unique reactivity of the thiol group underlies the growing interest in cysteine-based functional materials. In polymer science, thiol-mediated disulfide bonds have been widely employed to construct redox-responsive networks and dynamic crosslinked systems [7,8]. Moreover, disulfide chemistry enables mucoadhesive interactions through thiol–disulfide exchange reactions, while thiol groups can rapidly interact with metals and oxidative species, facilitating strong adhesion even under wet conditions [9,10]. Beyond disulfide bonding, thiol groups readily participate in Michael addition reactions with electron-deficient alkenes such as maleimide, methacrylate, and acrylamide, providing versatile routes for further functionalization [11,12]. Owing to these characteristics, cysteine-functionalized polymers have been extensively explored for antioxidant materials, redox-responsive hydrogels, and enhanced mucoadhesive systems [13,14,15]. Thiol-functionalized polysaccharides have recently emerged as an important class of bioactive polymers for biomedical applications [16,17,18]. However, to effectively integrate thiol-mediated functionalities into biomedical polymer applications, a structurally versatile and intrinsically biocompatible polymer platform is required.

Among various candidates, polysaccharides represent attractive natural polymer platforms owing to their high biocompatibility and structural diversity derived from distinct backbone compositions and functional groups [19,20]. As bio-based materials, polysaccharides offer environmental and biomedical advantages over synthetic polymers. Thiol-functionalized derivatives based on polysaccharides such as alginate, pectin, gellan gum, and hyaluronic acid have demonstrated high biocompatibility while exhibiting improved adhesiveness and antioxidant performance [21,22,23,24]. These previous studies indicated the potential of introducing thiol groups into polysaccharide backbones to impart additional functionality while preserving their inherent biological properties. Succinoglycan (SG), a microbial anionic polysaccharide, has attracted increasing attention as a promising polymer platform due to its high viscoelasticity, thermal stability, aqueous solubility, and biocompatibility [25,26]. These distinctive physicochemical properties, combined with its microbial origin and well-defined repeating unit structure, make SG a particularly attractive backbone for functional material development compared to other polysaccharides. Owing to the presence of carboxyl and hydroxyl groups, SG can undergo various chemical modifications [27,28,29]. In particular, EDC (1-ethyl-3-(3-dimethylaminopropyl)carbodiimide)/NHS (N-hydroxysuccinimide)-mediated coupling represents a widely applicable bioconjugation strategy, in which EDC activates carboxyl groups (–COOH) to form reactive O-acylisourea intermediates. However, these intermediates are unstable and prone to hydrolysis in aqueous conditions. NHS stabilizes these intermediates by converting them into NHS ester intermediates, which subsequently react with primary amine groups (–NH_2_) to form stable amide bonds. This chemistry enables the efficient introduction of functional moieties into the SG backbone while maintaining its intrinsic properties, and has been applied to cysteine conjugation in several other polysaccharides. Although such thiol-functionalized derivatives have been reported for alginate, hyaluronic acid, and related polysaccharides, thiol-functionalized derivatives of succinoglycan have not yet been reported. Such modifications are expected to expand the applicability of SG in hydrogel and film systems with enhanced mechanical and functional properties.

In this study, we hypothesized that the incorporation of cysteine via EDC/NHS-mediated amidation would introduce reactive thiol groups while preserving the intrinsic polysaccharide structure of SG. Such thiol functionalities were expected to enhance intermolecular interactions and redox activity, thereby improving the rheological properties and antioxidant performance of the resulting polymer [30,31,32]. Particularly, the introduction of thiol groups was expected to provide additional functional advantages in biomaterial systems, including redox-responsive behavior, improved intermolecular interactions through disulfide formation, and enhanced radical-scavenging capability. These properties are highly relevant in the design of antioxidant biomaterials, stimuli-responsive hydrogels, and drug delivery systems where redox activity and network adaptability play important roles.

To test this hypothesis, SG-Cys was synthesized and comprehensively characterized in terms of its structural, physicochemical, and functional properties to evaluate its potential as a multifunctional bio-based polymer. This study demonstrates that thiol functionalization of SG significantly enhances its rheological and antioxidant properties, highlighting its potential as a multifunctional bio-based polymer for applications in biomaterials, drug delivery, and bioengineering.

## 2. Materials and Methods

### 2.1. Materials

*Sinorhizobium meliloti* (*S. meliloti*) strain Rm 1021 was provided by the Microbial Carbohydrate Resource Bank (MCRB) at Konkuk University, Seoul, Republic of Korea. The HEK-293 cell line was provided by the Korean Cell Line Bank (Seoul, Republic of Korea). All chemical reagents were purchased from Sigma-Aldrich Chemicals Co. (St. Louis, MO, USA).

### 2.2. Culture Conditions and Isolation of Succinoglycan (SG)

Succinoglycan (SG) produced by *Sinorhizobium meliloti* Rm1021 was obtained following a previously described procedure with minor modification [33].

The bacterial strain was grown for 7 days at 30 °C under continuous agitation at 180 rpm in a culture medium containing D-mannitol (10 g/L), glutamic acid (1 g/L), K_2_HPO_4_ (1 g/L), MgSO_4_·7H_2_O (0.2 g/L), and CaCl_2_·2H_2_O (0.04 g/L), with the initial pH adjusted to 7.0. After incubation, the culture broth was centrifuged at 8000× *g* for 15 min at 4 °C to remove bacterial cells, and the supernatant containing the extracellular polysaccharide was collected. SG was recovered from the supernatant by ethanol precipitation using three volumes of ethanol. The precipitated polymer was subsequently dissolved in distilled water and purified by dialysis using a membrane with a molecular weight cut-off of 12–14 kDa against distilled water for 72 h. Finally, the purified SG solution was lyophilized to obtain dry SG for subsequent experiments.

### 2.3. Thiol Modification of SG (SG-Cys)

SG-Cys was prepared through the conjugation of L-cysteine to SG using EDC and NHS as coupling agents [34,35].

Initially, 0.5 g of succinoglycan was dispersed in 100 mL of purified water until a homogeneous solution was obtained. To activate the carboxyl functionalities of SG, EDC (2.8 mmol) together with NHS (1.4 mmol) was introduced into the solution, followed by stirring for 1 h. After the activation step, L-cysteine hydrochloride was added in different molar amounts (0.3, 0.6, 1.2, 2.4, and 4.8 mmol). The reaction medium was then adjusted to around pH 5.0 using aqueous NaOH or HCl solutions. The reaction was maintained at 25 °C for 12 h with continuous stirring under a nitrogen atmosphere. After the reaction, the mixture was dialyzed against dilute HCl (pH 3.5) (MWCO 12–14 kDa) at 25 °C for 2 days and then against deionized water for 1 day to remove residual acid. The resulting dialysate was freeze-dried using a vacuum freeze dryer. According to the amount of L-cysteine used, the products were designated as SG-Cys 0.5, SG-Cys 1, SG-Cys 2, SG-Cys 4, and SG-Cys 8.

The molecular weights and polydispersity indices (PDI) of the synthesized SG-Cys samples were determined by gel permeation chromatography (GPC) to confirm successful conjugation and to evaluate changes in polymer chain characteristics following thiol modification. GPC analysis was performed using a Waters Alliance e2695 system (Waters, Milford, MA, USA) equipped with a refractive index (RI) detector. Four columns (Waters Ultrahydrogel Linear, 500, 250, and 120) connected in series were used for separation. Pullulan standards with peak molecular weights of 642,000, 334,000, 201,000, 110,000, 49,400, 22,000, 9800, and 6300 g/mol were used for calibration. The elution solvent was 0.02 N sodium nitrate (NaNO_3_) solution at a flow rate of 0.8 mL/min and a column temperature of 35 °C.

### 2.4. Determination of Thiol and Disulfide Bond Content

The concentration of thiol groups in SG-Cys was determined using Ellman’s reagent (DTNB) with slight modification of a previously reported method [36]. For the assay, 0.2 mL of polymer dispersion (5 mg/mL) was diluted with 1.8 mL of phosphate buffer (0.5 M, pH 8.0). Subsequently, 2 mL of DTNB solution (0.03%, *w*/*v*), prepared in the same buffer, was introduced into the mixture. The reaction solution was kept at room temperature for 2 h before centrifugation. The absorbance of the resulting supernatant was recorded at 412 nm using a UV–visible spectrophotometer (Shimadzu, Kyoto, Japan). The thiol concentration was quantified using an L-cysteine calibration curve (R^2^ ≥ 0.99).

To evaluate the disulfide bond content, disulfide linkages in the polymer were first reduced to free thiol groups using sodium borohydride (NaBH_4_) [9,37]. In the reduction procedure, the polymer dispersion (15 mg/mL, 0.2 mL) was mixed with 1.3 mL of phosphate buffer (0.05 M, pH 6.8). Freshly prepared NaBH_4_ solution (2 mL, 4% *w*/*v*) was then added, and the mixture was incubated in a water bath at 37 °C for 1 h to complete the reduction. After the reduction step, residual NaBH_4_ was quenched by adding 0.5 mL of 5 M HCl. The reaction medium was subsequently neutralized with 2 mL of phosphate buffer (1 M, pH 8.5). The amount of thiol group was then determined following the aforementioned procedure. The experiments were performed in triplicate.

### 2.5. Structural Characterization of SG-Cys

#### 2.5.1. Fourier Transform Infrared (FTIR) Spectroscopy

Fourier transform infrared spectra of the freeze-dried samples were obtained using an FTIR spectrometer (Spectrum Two, PerkinElmer, Waltham, MA, USA). Measurements were carried out over a wavenumber range of 4000–650 cm^−1^ with a spectral resolution of 2 cm^−1^.

#### 2.5.2. Nuclear Magnetic Resonance (NMR) Spectroscopy

For NMR analysis, SG-Cys samples were dissolved in D_2_O to prepare a 1% (*w*/*v*) solution at 25 °C. The ^1^H NMR spectra were acquired using a Bruker Avance III 600 MHz spectrometer (Bruker, Karlsruhe, Germany).

#### 2.5.3. X-Ray Diffraction (XRD)

The crystalline structures of SG and SG-Cys were investigated by X-ray diffraction using a Rigaku SmartLab diffractometer (Rigaku, Akishima, Japan) with Cu Kα radiation. The instrument was operated at 40 kV and 30 mA, and diffraction patterns were collected over a 2θ range of 10–80°.

#### 2.5.4. Scanning Electron Microscope (SEM) Imaging

The surface morphologies of SG and SG-Cys were examined using a scanning electron microscope (FEI NanoSEM-450, Brno, Czech Republic). Micrographs were recorded at various magnifications under an accelerating voltage of 5 kV.

### 2.6. Thermogravimetric Analysis (TGA)

Thermal stability of SG and SG-Cys was evaluated by thermogravimetric analysis using a Discovery TGA 5500 instrument (TA Instruments, New Castle, DE, USA). Approximately 10 mg of each sample was heated from 30 to 600 °C at a constant heating rate of 10 °C min^−1^ under a nitrogen atmosphere.

### 2.7. Rheological Property Measurements

The rheological behavior of SG and SG-Cys solutions was evaluated using a DHR-2 rheometer (TA Instruments, New Castle, DE, USA) fitted with 60 mm parallel plates. Aqueous polymer solutions (1.0 wt%) were prepared in deionized water and analyzed at 25 °C unless otherwise stated. Strain sweep experiments were conducted to determine the linear viscoelastic region (LVE) of the samples by increasing the strain amplitude from 0.1% to 1000% at a constant angular frequency of 1 rad/s. Frequency sweep measurements were subsequently performed within the determined LVE range, with angular frequency varied from 0.1 to 100 rad/s at a fixed strain of 0.5%. Steady-shear viscosity was measured as a function of shear rate (0.1–1000 s^−1^). Thixotropy was evaluated using a hysteresis loop test by ramping the shear rate from 0.1 to 1000 s^−1^ and back, and yield stress was determined from shear stress–shear rate flow curves with each point held for 5 s prior to recording. Concentration-dependent rheology was assessed using SG and SG-Cys 4 solutions at 1.0, 1.5, 2.0, 2.5, and 3.0 wt%. Salt tolerance was evaluated by measuring viscosity in the presence of 0.25 M salt solutions (CaCl_2_, MgCl_2_, KCl, and NaCl), and pH stability was examined at pH 2, 5, 7, 9, and 12 using 1.0 wt% solutions. Temperature-dependent viscosity was evaluated at 25, 35, 45, 55, 65, and 75 °C, and temperature sweep tests were performed from 25 to 80 °C at a heating and cooling rate of 10 °C/min. Unless otherwise specified, viscosity in these tests was recorded over shear rates of 0.1–1000 s^−1^.

### 2.8. Antioxidant Activities

#### 2.8.1. ABTS Radical Scavenging Activity

The antioxidant activity of SG and SG-Cys was determined using the ABTS radical cation decolorization assay [38]. The ABTS radical solution was generated by combining equal volumes of 7 mM ABTS stock solution and 2.45 mM potassium persulfate (K_2_S_2_O_8_) followed by incubation of the mixture for 24 h at room temperature in the absence of light. Prior to use, the resulting solution was diluted with PBS buffer (pH 7.4) until an absorbance value of 0.70 ± 0.02 at 734 nm was obtained. For the assay, 500 μL of each sample solution was added to 3 mL of the prepared ABTS radical solution. The mixture was incubated at 37 °C for 8 min under dark conditions. After incubation, the absorbance was recorded at 734 nm using a UV–visible spectrophotometer. The ABTS radical scavenging activity was calculated according to Equation (1):

(1) $$ABTS\;radical\;scavenging\;activity\left(\%\right)=\frac{\left(A_{0}-A_{s}\right)}{A_{0}}\times 100$$

where *A*_0_ represents the absorbance of the ABTS radical solution without sample and *A_s_* corresponds to the absorbance measured after reaction with the sample. All measurements were performed in triplicate to ensure accuracy.

#### 2.8.2. DPPH Radical Scavenging Activity

The free radical scavenging activity of SG and SG-Cys was also evaluated using the DPPH method [39]. Various concentrations of SG or SG-Cys solution (1.0–3.0 mg/mL) were prepared, and 1 mL of each solution was mixed with 0.5 mL of DPPH ethanol solution (0.3 mM). The reaction mixtures were maintained at room temperature in the dark for 30 min. Ascorbic acid was used as a positive control. The DPPH radical scavenging activity was calculated according to Equation (2):

(2) $$DPPH\;radical\;scavenging\;activity\left(\%\right)=\frac{A_{c}-\left(A_{s}-A_{0}\right)}{A_{c}}\times 100$$

where *A_c_* denotes the absorbance of the control solution (1 mL deionized water mixed with 0.5 mL DPPH solution), *A_s_* represents the absorbance of the sample reaction mixture, and *A*_0_ indicates the absorbance of the sample solution prepared without DPPH. Each measurement was performed in triplicate.

### 2.9. Cytotoxicity Assay

The cytocompatibility of SG and SG-Cys was evaluated using an MTT assay with human embryonic kidney cells (HEK-293, Korean Cell Line Bank, Seoul, Republic of Korea) [40]. The cells were cultured in minimal essential medium (MEM, WELGENE, Republic of Korea) supplemented with 10% fetal bovine serum (FBS) and 1% penicillin–streptomycin. Cell suspensions were seeded into 96-well plates (Costar, Cambridge, MA, USA) at a density of 2.4 × 10^4^ cells/mL and allowed to attach under standard culture conditions (37 °C, 5% CO_2_). After sample treatment, cell viability was analyzed at 48 and 72 h intervals. The absorbance of each well was recorded at 570 nm using a SpectraMax microplate reader (Molecular Devices, San Jose, CA, USA) to quantify cell proliferation. The cell viability was calculated using Equation (3):

(3) $$Cell\;viability\left(\%\right)=\frac{\left(A_{s}\;-A0\right)}{\left(A_{c}-A0\right)}\times 100$$

where *A_s_* is the absorbance value of cells cultured with SG-Cys samples, *A_c_* is the absorbance value of control cells, and *A*_0_ is the blank solution containing minimal essential medium, MTT assay solution, and solubilization solution. Each test was performed in triplicate.

## 3. Results and Discussion

### 3.1. Characterization of SG-Cys

**Figure 1:**
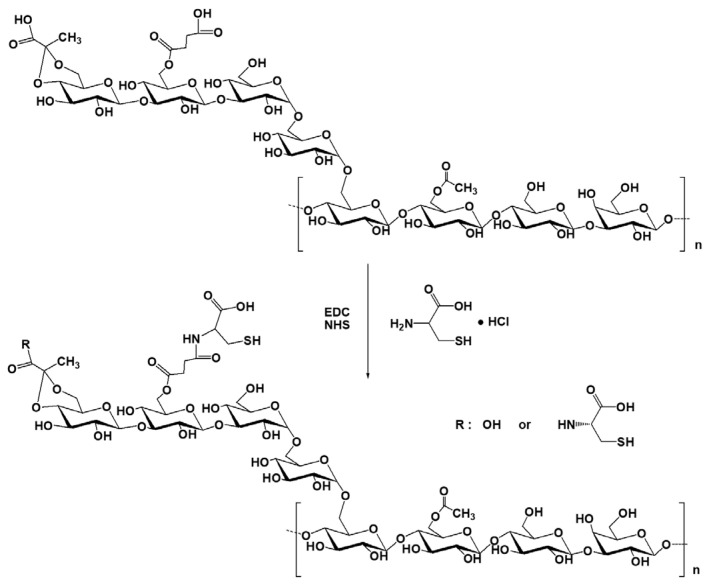
Schematic illustration of SG-Cys preparation.

The cysteine-modified succinoglycan (SG-Cys) was synthesized via EDC/NHS-mediated amide bond formation between the carboxyl groups of SG (pyruvic acid and succinic acid residues) and the amine group (–NH_2_) of L-cysteine, as illustrated in Figure 1.

To optimize the degree of thiol modification, five SG-Cys formulations (SG-Cys 0.5–8) were prepared by varying the molar ratio of SG(–COOH) to L-cysteine(–NH_2_) from 1:0.5 to 1:8, and their molecular weights, polydispersity indices (PDI), and reaction yields were summarized in Table 1.

SG showed a molecular weight of 330,800 g/mol with a narrow PDI of 1.459, as determined by gel permeation chromatography (GPC). After thiol modification, all SG-Cys samples exhibited a marked decrease in molecular weight (260,400–283,200 g/mol). No clear monotonic dependence of Mw or PDI on the L-cysteine feed ratio was observed. Similar Mw decreases after thiolation have been reported for other polysaccharides [38].

#### 3.1.1. Thiol Group and Disulfide Bond Content Determination

**Figure 2:**
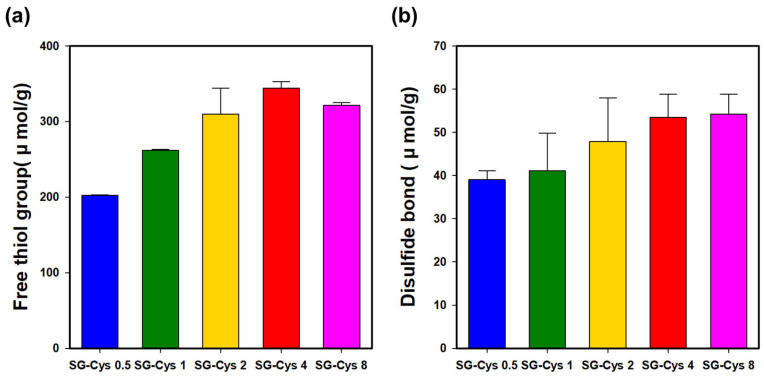
(**a**) Thiol group content and (**b**) disulfide bond content of SG-Cys (0.5–8).

As shown in Figure 2a, thiolation increased the thiol group content to 202.4–344.4 μmol/g across the formulations. The thiol content generally increased with increasing L-cysteine concentration; however, SG-Cys 8 showed a lower thiol content than SG-Cys 4. This trend can be attributed to the fixed stoichiometric amounts of EDC and NHS used for activating SG carboxyl groups, which inherently limit the number of carboxyl groups that can be converted to active ester intermediates [41]. When the L-cysteine feed ratio exceeds this activation capacity, the available activated intermediates are fully consumed and further conjugation is restricted, resulting in a plateau or a slight decrease in thiol content [38,42]. These results indicate that SG-Cys 4 represents the optimal formulation under the tested EDC/NHS reaction conditions.

The thiol content achieved in SG-Cys (up to 344.4 μmol/g) compares favorably with values reported for other thiolated polysaccharides. Thiolated low-methoxyl pectin has been reported to exhibit thiol contents in the range of 77.8–296 μmol/g, while thiolated cellulose derivatives have achieved thiol contents of approximately 215.5 μmol/g [9,43]. These results suggest that the EDC/NHS-mediated cysteine conjugation strategy employed in the present study is effective for introducing a high density of thiol groups onto microbial polysaccharides such as succinoglycan.

Thiol groups are susceptible to oxidation, which can lead to intermolecular disulfide bond formation. As shown in Figure 2b, the disulfide bond content ranged from 39.0 to 54.0 μmol/g, with no statistically significant differences among the samples (*p* > 0.05). Overall, approximately 23.6–37.8% of the grafted thiol groups in SG-Cys were oxidized to form disulfide crosslinks under the experimental conditions employed in this study [44]. The free thiol and disulfide bond contents for all SG-Cys formulations are summarized in Table 2.

#### 3.1.2. Fourier Transform Infrared (FTIR) Spectroscopy Analysis

**Figure 3:**
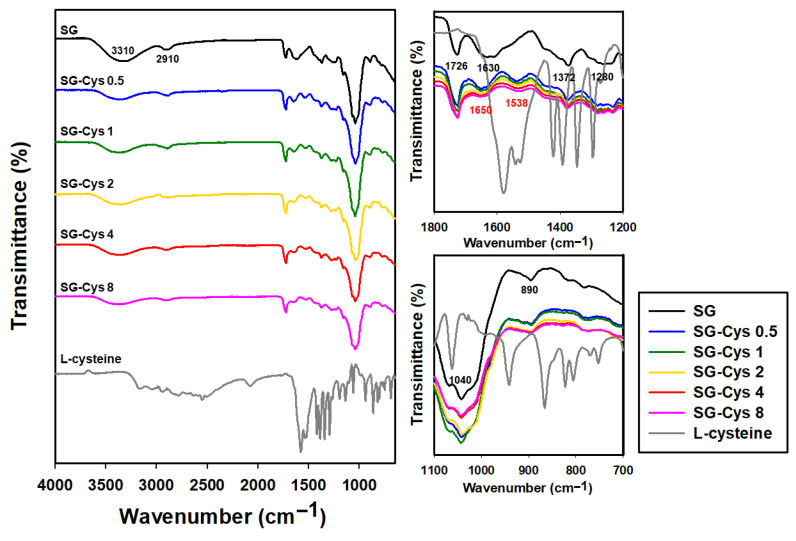
FTIR spectra of SG and SG-Cys (0.5–8).

FTIR spectroscopy was employed to investigate the structural changes between SG and SG-Cys conjugates. As shown in Figure 3, the FTIR spectra revealed characteristic absorption bands of the polysaccharide backbone. The broad absorption band at 3310 cm^−1^ was attributed to the stretching vibration of hydroxyl groups (–OH), while the peak at 2910 cm^−1^ corresponded to the asymmetric stretching vibrations of –CH_2_ and –CH_3_ groups inherent in polysaccharides. The carbonyl stretching vibration of carboxyl groups (C=O) appeared at 1726 cm^−1^, and the signal at 1630 cm^−1^ indicated the asymmetric stretching vibration of carboxylate groups. The symmetric stretching vibration of carboxylate groups (–COO^-^) from succinyl and pyruvyl substituents was identified at 1372 cm^−1^. Glycosidic linkage vibrations were evident through C–O–C stretching at 1280 cm^−1^ and asymmetric stretching at 1040 cm^−1^. The β-glycosidic bond stretching vibration was detected at 890 cm^−1^ [45,46].

Compared with the spectrum prior to amidation, SG-Cys exhibited peaks at 1650 and 1538 cm^−1^. The peak at 1650 cm^−1^ was assigned to amide I, corresponding to the C=O stretching vibration of the amide bond (O=C–NH–). In addition, the peak at 1538 cm^−1^ was attributed to amide II vibrations, arising from N–H in-plane deformation coupled with C–N stretching. The presence of these amide-related peaks suggested the formation of amide linkages between the carboxyl groups of SG and the amino group of cysteine, supporting successful covalent conjugation of cysteine onto the polysaccharide backbone [47,48].

#### 3.1.3. Nuclear Magnetic Resonance (NMR) Spectroscopy Analysis

**Figure 4:**
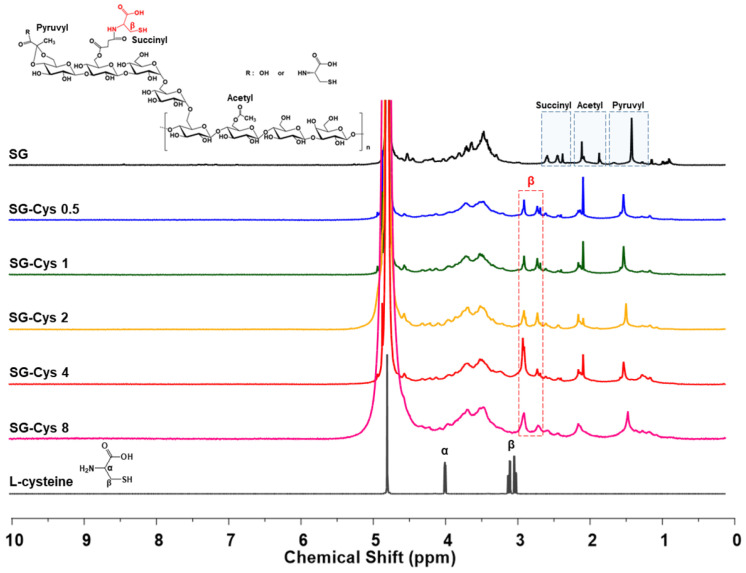
^1^H NMR spectra of SG and SG-Cys (0.5–8). D_2_O was used as the solvent.

The ^1^H NMR spectra of SG and SG-Cys are shown in Figure 4. SG exhibited broad carbohydrate peaks in the 3.2–4.8 ppm region, consistent with the polysaccharide backbone. In addition, peaks at 1.49, 2.10, and 2.59 ppm were assigned to the methyl protons of pyruvyl substituents, the methyl protons of acetyl groups (–COCH_3_), and the methylene protons of succinyl groups (–COCH_2_CH_2_COO^−^), respectively [49].

After L-cysteine modification, all SG-Cys samples retained the characteristic peaks of SG, indicating that the polysaccharide framework was preserved. New peaks at 2.73 and 2.92 ppm were observed in all SG-Cys samples and were absent in SG; these peaks were consistent with the methylene (β-CH_2_) protons of the cysteine residue (–CH_2_–SH). The α-CH proton of L-cysteine has been reported to resonate at 3.9–4.2 ppm; however, in the present spectra, this signal could not be clearly resolved due to overlap with the broad carbohydrate peaks in the 3.2–4.8 ppm region. Overall, these observations provided evidence for successful L-cysteine conjugation to SG [50,51].

#### 3.1.4. X-Ray Diffraction (XRD) Analysis

**Figure 5:**
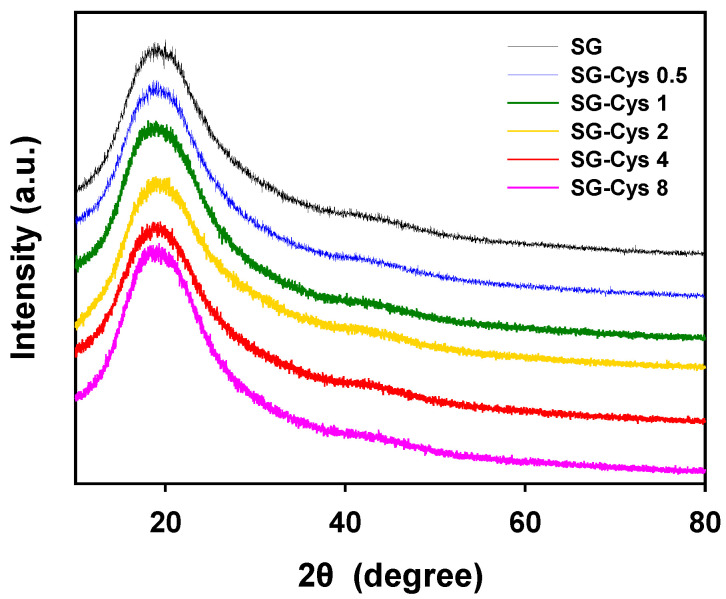
XRD patterns of SG and SG-Cys (0.5–8) measured at 25 °C.

X-ray diffraction (XRD) analysis was performed to examine the solid-state structure of SG and SG-Cys, as shown in Figure 5. All samples exhibited a broad diffraction halo centered at 2θ ≈ 20°, indicating a predominantly amorphous structure. The overall diffraction profiles of SG-Cys were comparable to that of native SG, with no distinct new crystalline reflections after L-cysteine modification. These results suggested that L-cysteine conjugation did not markedly alter the bulk solid-state structure of SG [52,53,54].

#### 3.1.5. Morphological Analysis of SG-Cys

**Figure 6:**
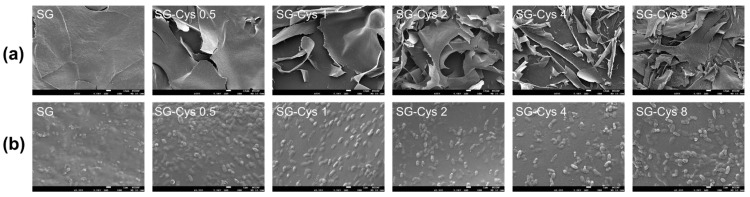
FE-SEM images of SG and SG-Cys (0.5–8). The scale bars are (**a**) 10 μm and (**b**) 1 μm, respectively.

The morphology of SG and SG-Cys was examined by FE-SEM, as shown in Figure 6. In Figure 6a, SG exhibited relatively large, sheet-like fragments, whereas SG-Cys samples displayed a more fragmented and irregular morphology with smaller debris-like features. This observation agreed well with the reduced molecular weight reported in Table 1 [38]. At higher magnification in Figure 6b, SG-Cys exhibited a rougher, more textured surface compared with SG. Similar surface texture changes have been reported for other thiol-modified polysaccharides [55]. This change in surface texture may have been associated with disulfide linkages introduced upon thiolation [56]. Overall, these morphological differences relative to SG supported the successful thiol modification of SG.

### 3.2. Thermal Properties Analysis

**Figure 7:**
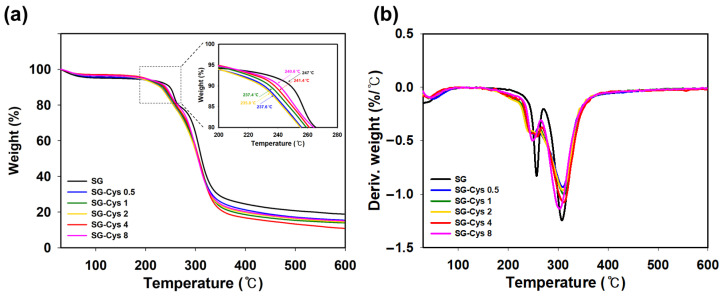
(**a**) TGA curves and (**b**) DTG curves of SG and SG-Cys (0.5–8).

The thermal properties of SG-Cys were evaluated by thermogravimetric analysis (TGA) as thermal stability is closely related to processability and potential industrial applications. TGA was used to examine mass-loss behavior upon heating under a nitrogen atmosphere. As shown in Figure 7a, the samples exhibited a multi-step thermal degradation behavior that could be described in three stages. The first stage showed a minor weight loss at low temperatures, mainly attributed to the removal of physically bound moisture. The second stage displayed a pronounced weight reduction at intermediate temperatures, corresponding to the primary thermal decomposition of the polysaccharide structure. The onset temperature of the main degradation, determined from the TGA, was 247 °C for SG, while SG-Cys derivatives exhibited lower onset temperatures (235.8–241.4 °C), indicating decreased thermal stability after thiolation [32]. The third stage involved a gradual mass decrease at higher temperatures, resulting in residual mass formation [57]. These results may have indicated that thiolation affected intermolecular interactions and the thermal transition behavior of SG. As shown in Figure 7b, SG-Cys derivatives exhibited broader and less pronounced DTG peaks at approximately 260 °C and 310 °C compared with native SG. This more gradual weight-loss behavior suggested that thermal degradation occurred over a wider temperature range, possibly due to enhanced intermolecular interactions induced by cysteine incorporation. Importantly, below 200 °C, the thermal stability of the SG backbone was largely preserved, suggesting that cysteine modification did not significantly compromise the intrinsic structural integrity of SG under moderate thermal conditions [58,59]. Considering that typical biomedical and processing applications operate well below this temperature range, the observed reduction in degradation temperature was unlikely to affect practical usability.

### 3.3. Rheological Property Measurement

#### 3.3.1. Oscillatory Shear Rheology

**Figure 8:**
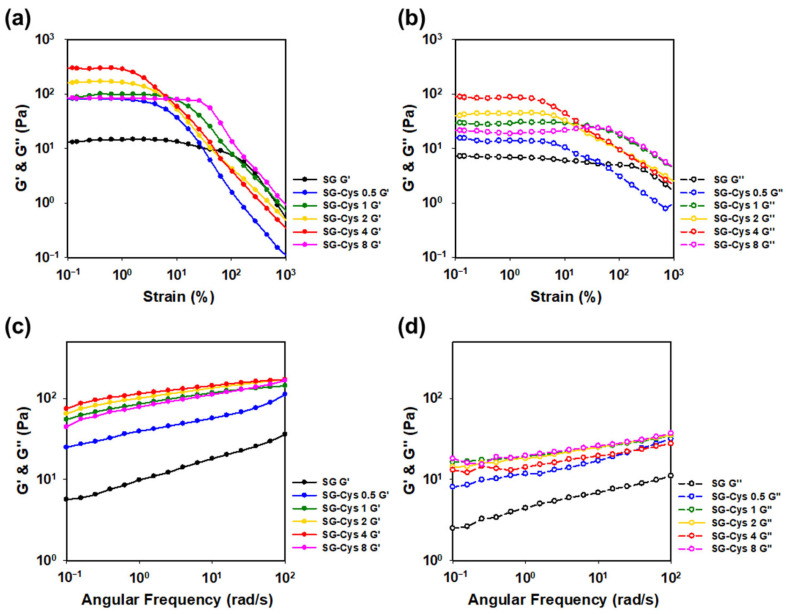
(**a**) Storage modulus (G′) and (**b**) loss modulus (G″) from amplitude sweep tests, and (**c**) storage modulus (G′) and (**d**) loss modulus (G″) from frequency sweep tests of SG and SG-Cys (0.5–8).

The rheological properties of SG and SG-Cys were evaluated by amplitude and frequency sweep measurements. As shown in Figure 8a,b, both SG and SG-Cys exhibited weak gel-like behavior in the linear viscoelasticity (LVE) region, with the storage modulus (G′) higher than the loss modulus (G″). Notably, SG-Cys showed higher G′ and G″ values than SG, and both moduli increased with increasing L-cysteine substitution degree (thiol content), indicating a strengthened network at small deformations [32].

With increasing strain, SG maintained an elastic-dominant response (G′ > G″) over a wider strain range, whereas SG-Cys samples exhibited an earlier G′–G″ crossover at lower strains [41]. This shift to a lower crossover strain suggested reduced resistance to large deformations, likely due to disruption and rearrangement of the gel/network structure under strain, leading to a transition from elastic- to viscous-dominant behavior.

Based on the amplitude sweep results, 0.5% strain was within the LVE region for all samples; accordingly, the frequency sweep was performed at 0.5% strain. As shown in Figure 8c,d, SG and SG-Cys maintained a dominant elastic response (G′ > G″) over the measured angular frequency range. Moreover, the progressive increase in both G′ and G″ with thiol content represented the formation of a stronger and more elastic network, which may have been associated with improved structural stabilization via intra- and intermolecular thiol/disulfide-mediated interactions, including free thiol associations and disulfide crosslinking [60,61].

#### 3.3.2. Steady-Shear Flow Behavior

**Figure 9:**
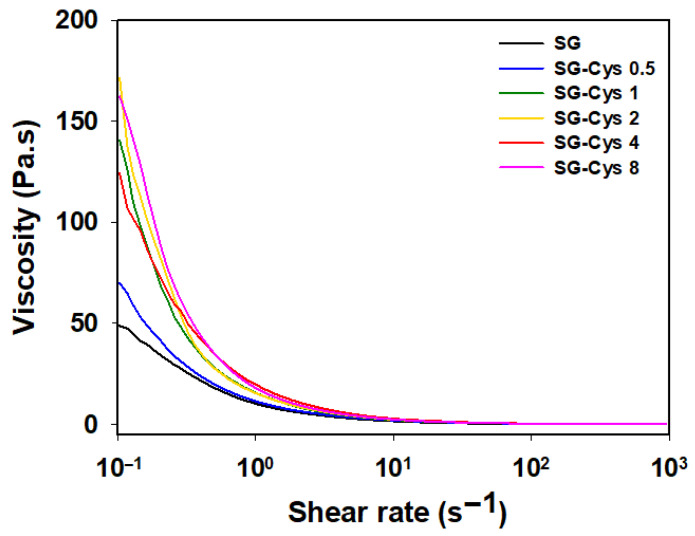
Shear viscosity as a function of shear rate for SG and SG-Cys (0.5–8).

The viscosity properties of SG and SG-Cys were evaluated at a concentration of 1.0 wt%. As shown in Figure 9, SG-Cys exhibited higher viscosity than SG across the tested conditions [62]. At a shear rate of 10 s^−1^, the viscosity of SG was 1.58 Pa·s, whereas the viscosities of SG-Cys 0.5, SG-Cys 1, SG-Cys 2, SG-Cys 4, and SG-Cys 8 were 2.05, 2.34, 2.45, 2.89, and 2.50 Pa·s, respectively. Overall, viscosity tended to increase with increasing L-cysteine substitution degree (free thiol content) [41,43]. In particular, SG-Cys 4 showed the greatest enhancement, reaching ~1.83-fold higher viscosity than SG. The observed increase in viscosity with higher L-cysteine substitution was consistent with previously reported trends in polysaccharide rheology, where structural modifications that enhance intermolecular interactions and effective polymer volume fraction lead to increased solution viscosity [63]. The slight decrease in viscosity observed for SG-Cys 8 is attributable to its reduced free thiol content relative to SG-Cys 4, which diminished the extent of intermolecular thiol-mediated interactions.

**Figure 10:**
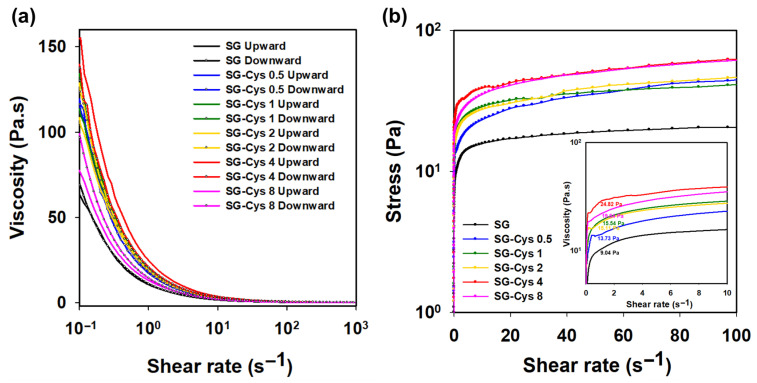
(**a**) Thixotropic behavior of SG and SG-Cys (0.5–8) assessed by an up–down shear-rate cycle (0.1–1000 s^−1^). (**b**) Yield stress analysis of SG and SG-Cys (0.5–8), defined at the onset of flow.

Thixotropic behavior was assessed using an alternating up–down shear-rate cycle test (Figure 10a). All samples showed minimal hysteresis between the upward and downward sweeps, suggesting rapid structural recovery and weak thixotropy [64].

In addition, the yield stress was analyzed (Figure 10b). The yield stress was defined as the stress at the onset of flow, i.e., where the shear rate began to increase [65]. SG exhibited a yield stress of 9.04 Pa, whereas all SG-Cys samples showed higher yield stresses than SG. Among them, SG-Cys 4 exhibited the highest yield stress of 24.82 Pa. The increase in yield stress with higher degrees of thiolation was consistent with previous reports indicating that enhanced intermolecular interactions and network connectivity in polymer systems contribute to higher resistance against flow, leading to elevated yield stress as a result of a denser or more entangled polymer network [66]. These results suggested that yield stress increased with increasing degree of thiolation, consistent with the viscosity results, indicating improved rheological performance of SG-Cys 4. Therefore, SG-Cys 4 was selected as the optimal formulation for subsequent experiments comparing rheological properties with SG.

#### 3.3.3. Viscosity Characterization Across Concentration and External Conditions

The rheological properties of polysaccharides are key indicators for evaluating their potential as industrial ingredients, including thickeners, stabilizers, texture modifiers, and gelling agents, and are therefore closely associated with commercial applicability [67,68]. For industrial utilization, it is essential to develop biomaterials that maintain stable viscosity under diverse environmental conditions, such as concentration, ionic strength/salt type, pH, and temperature. Accordingly, the viscosity of SG and SG-Cys 4 was systematically evaluated under varying concentration, salt type, pH, and temperature conditions.

**Figure 11:**
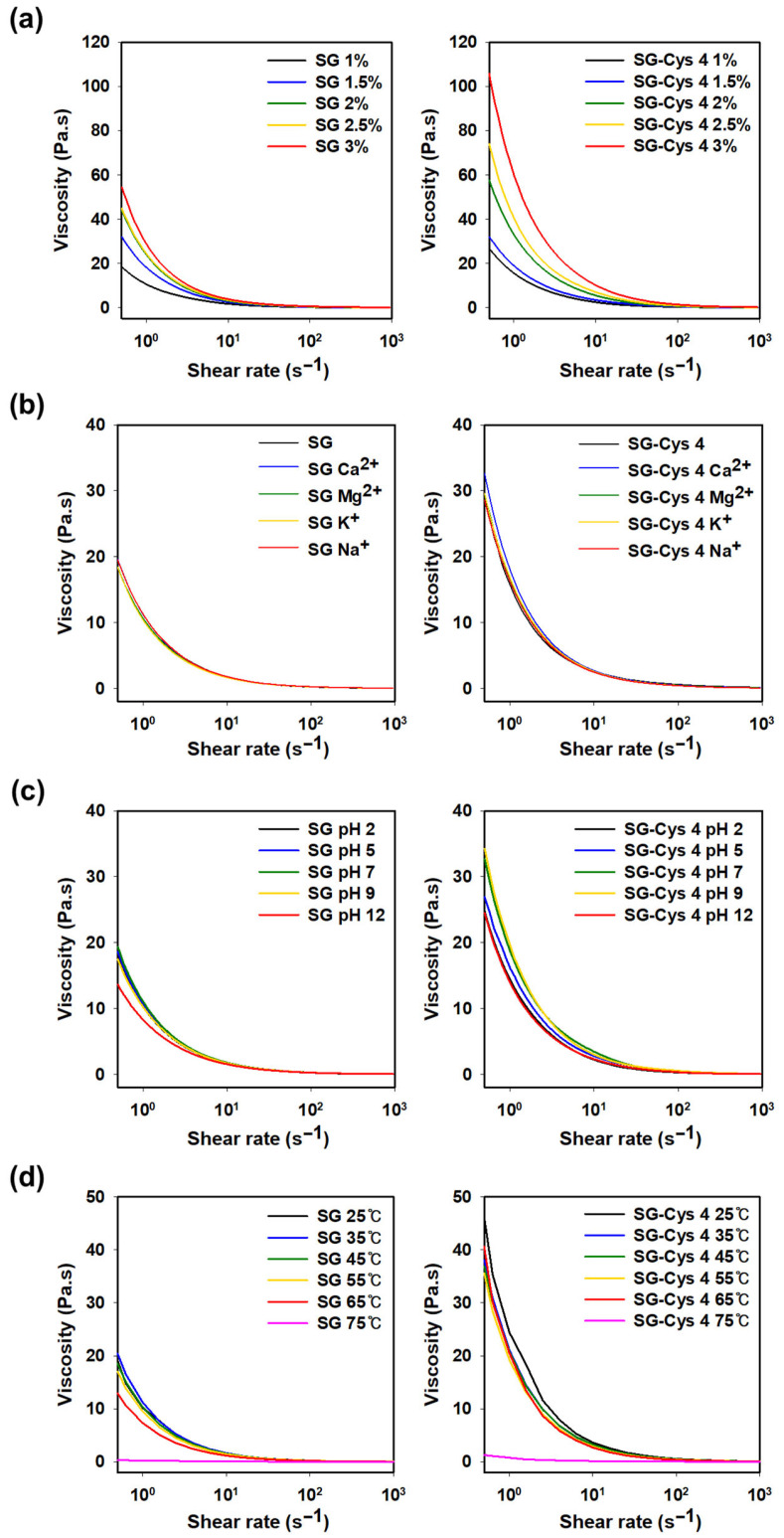
Viscosity of SG and SG-Cys 4 as a function of (**a**) concentration, (**b**) salt type, (**c**) pH, and (**d**) temperature.

Figure 11a showed the viscosity profiles of SG and SG-Cys 4 solutions as the polymer concentration was increased from 1 to 3 wt%. A concentration-dependent increase in viscosity was observed for both samples. At a shear rate of 10 s^−1^, the viscosity of 3 wt% SG was 3.83 Pa·s, whereas that of 3 wt% SG-Cys 4 reached 10.17 Pa·s. Notably, SG-Cys 4 consistently displayed higher viscosity than SG at all concentrations, suggesting that thiolation may have enhanced intermolecular interactions.

Figure 11b presented viscosities measured in the presence of 0.25 M salts (CaCl_2_, MgCl_2_, KCl, and NaCl) [69,70]. Both SG and SG-Cys 4 showed no notable change in viscosity under these ionic conditions, indicating that their viscosity was maintained even at relatively high ionic strength and in the presence of different cations.

Figure 11c summarized the pH-dependent viscosity behavior. At a shear rate of 10 s^−1^, SG exhibited viscosities of 1.65, 1.72, 1.77, 1.70, and 1.54 Pa·s at pH 2.0, 5.0, 7.0, 9.0, and 12.0, respectively, corresponding to a viscosity variation of <13.0% over pH 2.0–12.0. Under the same conditions, SG-Cys 4 showed viscosities of 2.23, 2.79, 3.44, 3.01, and 2.31 Pa·s at pH 2.0, 5.0, 7.0, 9.0, and 12.0, respectively, indicating greater pH responsiveness than SG. In particular, the viscosity increased from pH 2.0 to 7.0, reached a maximum at pH 7.0, and then decreased at pH 9.0 and 12.0. This trend was consistent with previously reported results for thiolated chitosan [56]. Under acidic conditions, most thiol groups remained in the reduced –SH form, and the formation of intermolecular disulfide bonds was limited, resulting in relatively weak intermolecular interactions within the polymer network. The maximum viscosity near pH 7.0 may be attributed to partial oxidation of thiol groups introduced into SG-Cys 4, leading to disulfide bond (S–S) formation and increased interchain crosslinking, which can effectively increase network connectivity and chain entanglement. In contrast, under alkaline conditions above pH 7.0, base-catalyzed degradation of polysaccharide chains (chain scission/depolymerization) may have been promoted, resulting in a reduced average molecular weight and, consequently, decreased viscosity. Nevertheless, SG-Cys 4 maintained viscosities above 2.2 Pa·s across the entire pH range tested (pH 2.0–12.0), indicating robust viscosity retention.

Figure 11d showed the temperature-dependent viscosity of SG and SG-Cys 4 measured from 25 to 75 °C. Both samples maintained relatively stable viscosity up to 65 °C, indicating that their solution viscosity was largely preserved within this temperature window; however, a pronounced decrease was observed at 75 °C. This trend was consistent with thermally induced weakening of intermolecular associations and reduced flow resistance. For SG, the viscosity loss at elevated temperatures was associated with relaxation of its helical molecular conformation.

**Figure 12:**
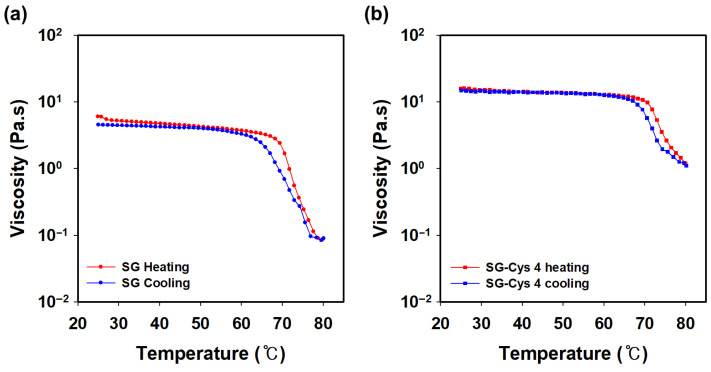
Temperature-dependent viscosity of (**a**) SG and (**b**) SG-Cys 4 over a heating–cooling cycles from 25 °C to 80 °C at a shear rate of 1 s^−1^.

Figure 12 showed the viscosity response observed over a heating–cooling cycle between 25 and 80 °C. As the temperature increased, both samples exhibited a gradual reduction in viscosity. At 80 °C, SG decreased to approximately 0.08 Pa·s, whereas SG-Cys 4 retained a substantially higher viscosity of about 1.2 Pa·s. Upon subsequent cooling, both samples exhibited viscosity recovery, to values comparable to their initial state at 25 °C. These results suggest that the viscosity loss over 25–80 °C is largely reversible and primarily governed by reversible changes in intermolecular interactions, with limited irreversible degradation under the tested conditions. The reversible decrease in viscosity upon heating is likely associated with temperature-induced weakening of intermolecular interactions such as hydrogen bonding and polymer chain entanglements [71,72]. The higher viscosity retained by SG-Cys suggests that thiol-mediated intermolecular associations, including possible disulfide interactions, contribute to enhanced network stability.

### 3.4. Antioxidant Activity

**Figure 13:**
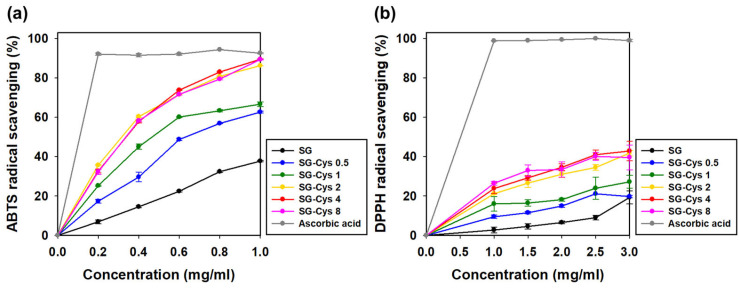
(**a**) ABTS and (**b**) DPPH radical scavenging activities of SG and SG-Cys (0.5–8) at different concentrations. Ascorbic acid was used as a positive control. Data are presented as mean ± SD (*n* = 3).

The antioxidant activity of SG and SG-Cys samples was evaluated using ABTS and DPPH radical scavenging assays. As shown in Figure 13a, ABTS radical scavenging activities were measured at sample concentrations ranging from 0.2 to 1.0 mg/mL. At 1.0 mg/mL, the ABTS radical scavenging activities of SG, SG-Cys 0.5, SG-Cys 1, SG-Cys 2, SG-Cys 4, SG-Cys 8, and ascorbic acid were 37.71%, 62.61%, 66.65%, 86.22%, 89.43%, 89.36%, and 92.54%, respectively. As shown in Figure 13b, DPPH radical scavenging activities were measured at sample concentrations ranging from 1.0 to 3.0 mg/mL. At 3.0 mg/mL, the DPPH radical scavenging activities of SG, SG-Cys 0.5, SG-Cys 1, SG-Cys 2, SG-Cys 4, SG-Cys 8, and ascorbic acid were 19.26%, 19.72%, 27.21%, 41.79%, 42.86%, 39.56%, and 98.94%, respectively.

Overall, thiol introduction markedly improved the radical scavenging capacity of SG, and the antioxidant activity generally increased with increasing free thiol content as quantified in Figure 2 [39,73]. This enhancement could be attributed to the intrinsic redox activity of thiol groups (–SH), which are capable of donating hydrogen atoms or electrons to neutralize radical species via hydrogen atom transfer (HAT) or electron transfer (ET) mechanisms [74]. During this process, thiyl radicals (RS·) may form and subsequently undergo coupling reactions to generate disulfide bonds, thereby stabilizing the radical species and terminating chain reactions [75]. These well-established thiol-mediated redox reactions likely accounted for the enhanced ABTS and DPPH scavenging activities observed in SG-Cys derivatives.

### 3.5. Cell Cytotoxicity

**Figure 14:**
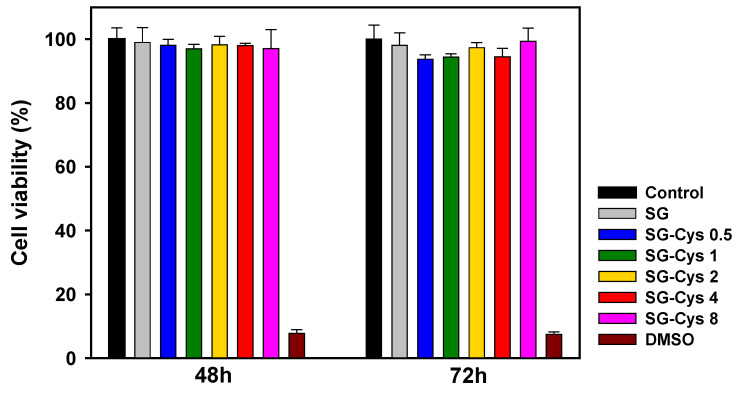
Cytotoxicity of SG and SG-Cys (0.5–8), as determined by an MTT assay in HEK293 cells after 48 and 72 h of incubation. Data are presented as mean ± SD (*n* = 3).

Cell cytotoxicity of SG and SG-Cys samples was evaluated in HEK-293 cells using an MTT assay kit. HEK-293 cells were treated with SG or SG-Cys at a concentration of 0.5 mg/mL, and DMSO-treated cells were used as the negative control. As shown in Figure 14, no apparent cytotoxicity was observed for any SG or SG-Cys sample. After 48 h of incubation, the cell viabilities of SG, SG-Cys 0.5, SG-Cys 1, SG-Cys 2, SG-Cys 4, and SG-Cys 8 were 98.99%, 98.11%, 96.94%, 98.18%, 97.98%, and 97.02%, respectively. After 72 h, the corresponding viabilities were 98.03%, 93.71%, 94.40%, 97.32%, 94.46%, and 99.34%, respectively. Therefore, these results indicated that SG and SG-Cys samples exhibited no cytotoxicity toward HEK-293 cells under the tested conditions [39,76,77].

## 4. Conclusions

In this study, cysteine-modified succinoglycan (SG-Cys) was successfully synthesized via EDC/NHS-mediated amidation, introducing thiol functionalities while preserving the intrinsic polysaccharide structure of succinoglycan. To the best of our knowledge, this study represents the first report of cysteine-mediated thiol functionalization of succinoglycan. Structural characterization by FTIR and ^1^H NMR confirmed the successful covalent conjugation of cysteine, and the thiol content reached up to 344.4 μmol g^−1^ depending on the feed ratio of cysteine. The incorporation of cysteine significantly altered the physicochemical and rheological properties of SG. In particular, SG-Cys derivatives exhibited higher viscosity, increased yield stress, and enhanced viscoelastic behavior compared with native SG. These improvements are likely associated with enhanced intermolecular interactions and partial disulfide bond formation between thiol groups, which may contribute to the formation of a more interconnected polymer network.

Furthermore, the introduction of thiol functionalities markedly enhanced the antioxidant capacity of SG-Cys, as demonstrated by ABTS and DPPH radical scavenging assays, while maintaining high cytocompatibility in HEK-293 cells.

From a biomaterials perspective, thiol-functionalized polymers have attracted increasing attention due to their ability to form dynamic disulfide networks, respond to redox environments, and interact with biological systems through thiol–disulfide exchange reactions. In this context, the SG-Cys platform developed in this study may provide a useful strategy for designing antioxidant biomaterials, bioactive hydrogels, and redox-responsive drug delivery systems.

Overall, these results demonstrate that cysteine conjugation provides an effective strategy for introducing thiol functionality into microbial polysaccharides and tailoring their physicochemical and biological properties. The resulting SG-Cys represents a promising thiol-functionalized bio-based polymer platform with potential applications in antioxidant biomaterials, bioactive hydrogels, and drug delivery systems.

## Figures and Tables

**Table 1:** Molecular weight, polydispersity and reaction yield of SG-Cys (0.5–8). Molecular weight was determined by gel permeation chromatography (GPC).

Sample	Ratio of SG(–COOH)/L-Cysteine(-NH_2_)	Molecular Weight (g/mol)	Polydispersity	Yield (%)
SG	Control	330,800	1.459	-
SG-Cys 0.5	1:0.5	273,200	1.468	96.6
SG-Cys 1	1:1	268,300	1.458	95.8
SG-Cys 2	1:2	283,200	1.425	95.0
SG-Cys 4	1:4	260,400	1.395	94.6
SG-Cys 8	1:8	265,900	1.402	91.6

**Table 2:** The content of the free thiol group and disulfide bond of SG-Cys prepared with different L-cysteine feed ratios.

	Polymer					
	SG	SG-Cys 0.5	SG-Cys 1	SG-Cys 2	SG-Cys 4	SG-Cys 8
Free thiol group (μmol/g)	-	202.4 ± 0.9	262.0 ± 1.2	310 ± 34	344.4 ± 8.4	321.5 ± 3.5
Disulfide bond (μmol/g)	-	39.0 ± 2.0	41.1 ± 8.7	48 ± 10	53.5 ± 5.3	54 ± 4.5

## Data Availability

The original contributions presented in this study are included in the article. Further inquiries can be directed to the corresponding author.

## Abbreviations

ABTS	2,2′-azinobis(3-ethylbenzothiazoline-6-sulfonic acid)
Cys	L-cysteine
DMSO	dimethyl sulfoxide
DPPH	2,2-diphenyl-1-picrylhydrazyl
DTG	derivative thermogravimetry
DTNB	5,5′-dithiobis(2-nitrobenzoic acid) (Ellman’s reagent)
EDC	1-ethyl-3-(3-dimethylaminopropyl)carbodiimide
ET	electron transfer
FTIR	Fourier transform infrared (spectroscopy)
G′	storage modulus
G″	loss modulus
GPC	gel permeation chromatography
HAT	hydrogen atom transfer
HEK-293	human embryonic kidney 293 cells
LVE	linear viscoelasticity
MTT	3-(4,5-dimethylthiazol-2-yl)-2,5-diphenyltetrazolium bromide
MWCO	molecular weight cut-off
Mw	weight-average molecular weight
NHS	N-hydroxysuccinimide
NMR	nuclear magnetic resonance
PBS	phosphate-buffered saline
PDI	polydispersity index
RI	refractive index
SEM	scanning electron microscopy
SG	succinoglycan
SG-Cys	cysteine-modified succinoglycan
TGA	thermogravimetric analysis
XRD	X-ray diffraction

## References

[1] Chrószcz-Porębska M., Gadomska-Gajadhur A. (2024). Cysteine conjugation: An approach to obtain polymers with enhanced muco-and tissue adhesion. Int. J. Mol. Sci..

[2] Thompson M., Scholz C. (2021). Highly branched polymers based on poly (amino acid) s for biomedical application. Nanomaterials.

[3] Paglia E.B., de Freitas G.P., Baldin E.K.K., Pacheco J.E.C., Carvalho H.F., Beppu M.M. (2025). Innovative approaches in alginate grafting: Amino acids of RGD peptide composition for biomimetic and biocompatible biomaterial. Int. J. Biol. Macromol..

[4] Zhang Q.-F., Luan C.-R., Yin D.-X., Zhang J., Liu Y.-H., Peng Q., Xu Y., Yu X.-Q. (2015). Amino acid-modified polyethylenimines with enhanced gene delivery efficiency and biocompatibility. Polymers.

[5] Gavriel K., Neumann K. (2026). Toward Precision Bioconjugation: Chemical Strategies for Site-Selective Cysteine Conjugation. Precis. Chem..

[6] Choi H., Kim M., Jang J., Hong S. (2020). Visible-Light-Induced Cysteine-Specific Bioconjugation: Biocompatible Thiol–Ene Click Chemistry. Angew. Chem. Int. Ed..

[7] Altinbasak I., Kocak S., Sanyal R., Sanyal A. (2022). Fast-forming dissolvable redox-responsive hydrogels: Exploiting the orthogonality of thiol–maleimide and thiol–disulfide exchange chemistry. Biomacromolecules.

[8] Ranamalla S.R., Tavakoli S., Porfire A.S., Tefas L.R., Banciu M., Tomuța I., Varghese O.P. (2024). A quality by design approach to optimise disulfide-linked hyaluronic acid hydrogels. Carbohydr. Polym..

[9] Kali G., Ozkahraman B., Laffleur F., Knoll P., Wibel R., Zoller K., Bernkop-Schnurch A. (2023). Thiolated cellulose: A dual-acting mucoadhesive and permeation-enhancing polymer. Biomacromolecules.

[10] Bernkop-Schnürch A. (2005). Thiomers: A new generation of mucoadhesive polymers. Adv. Drug Delivery Rev..

[11] Nair D.P., Podgórski M., Chatani S., Gong T., Xi W., Fenoli C.R., Bowman C.N. (2014). The thiol-Michael addition click reaction: A powerful and widely used tool in materials chemistry. Chem. Mater..

[12] Jansen L.E., Negrón-Piñeiro L.J., Galarza S., Peyton S.R. (2018). Control of thiol-maleimide reaction kinetics in PEG hydrogel networks. Acta Biomater..

[13] Budai-Szűcs M., Horvát G., Gyarmati B., Szilágyi B.Á., Szilágyi A., Berkó S., Ambrus R., Szabó-Révész P., Sandri G., Bonferoni M.C. (2017). The effect of the antioxidant on the properties of thiolated poly (aspartic acid) polymers in aqueous ocular formulations. Eur. J. Pharm. Biopharm..

[14] Bolanta S.O., Malijauskaite S., McGourty K., O’Reilly E.J. (2022). Synthesis of poly (acrylic acid)-cysteine-based hydrogels with highly customizable mechanical properties for advanced cell culture applications. ACS Omega.

[15] Park H.W., Kim J.-D. (2009). Mucoadhesive interaction of cysteine grafted poly (2-hydroxyethyl aspartamide) with pig mucin layer of surface plasmon resonance biosensor. J. Ind. Eng. Chem..

[16] Summonte S., Racaniello G.F., Lopedota A., Denora N., Bernkop-Schnürch A. (2021). Thiolated polymeric hydrogels for biomedical application: Cross-linking mechanisms. J. Control. Release.

[17] Huang Z.-J., Ye M.-N., Peng X.-H., Gui P., Cheng F., Wang G.-H. (2025). Thiolated chitosan hydrogel combining nitric oxide and silver nanoparticles for the effective treatment of diabetic wound healing. Int. J. Biol. Macromol..

[18] Taghizadeh F., Mehryab F., Mortazavi S.A., Rabbani S., Haeri A. (2023). Thiolated chitosan hydrogel-embedded niosomes: A promising crocin delivery system toward the management of aphthous stomatitis. Carbohydr. Polym..

[19] Rumon M.M.H., Akib A.A., Sarkar S.D., Khan M.A.R., Uddin M.M., Nasrin D., Roy C.K. (2024). Polysaccharide-based hydrogels for advanced biomedical engineering applications. ACS Polym. Au.

[20] Sousa V., Monteiro L.P., Rocha D.H., Rodrigues J.M., Borges J., Mano J.F. (2025). Marine-Origin Polysaccharides and Their Chemically Modified Derivatives as Sources of Advanced Biofunctional Materials for Biomedical Applications. Biomacromolecules.

[21] Sharma R., Ahuja M. (2011). Thiolated pectin: Synthesis, characterization and evaluation as a mucoadhesive polymer. Carbohydr. Polym..

[22] Chen T.C., Tang R.C., Lin J.N., Kuo W.T., Yang I.H., Liang Y.J., Lin F.H. (2023). The synthesis and evaluation of thiolated alginate as the barrier to block nutrient absorption on small intestine for body-weight control. Bioeng. Transl. Med..

[23] Abedini E., Shajari G., Barzegari A., Mahdipour M., Fathi M., Erfan-Niya H. (2026). Thiolated gellan gum/polyethylene glycol diacrylate hydrogel containing secretome as a wound dressing: In vitro and in vivo studies. Mater. Today Commun..

[24] Shu X.Z., Liu Y., Luo Y., Roberts M.C., Prestwich G.D. (2002). Disulfide cross-linked hyaluronan hydrogels. Biomacromolecules.

[25] Kim J., Jeong J.-P., Kim Y., Jung S. (2024). Physicochemical and rheological properties of succinoglycan overproduced by Sinorhizobium meliloti 1021 mutant. Polymers.

[26] Jeong J.-P., Kim Y., Hu Y., Jung S. (2022). Bacterial succinoglycans: Structure, physical properties, and applications. Polymers.

[27] Yang Y., Zhang H., Zhang X., Shen S., Wu B., Peng D., Yin J., Wang Y. (2025). A Succinoglycan-riclin-zinc-phthalocyanine-based composite hydrogel with enhanced photosensitive and antibacterial activity targeting biofilms. Gels.

[28] Shin Y., Hu Y., Park S., Jung S. (2023). Novel succinoglycan dialdehyde/aminoethylcarbamoyl-β-cyclodextrin hydrogels for pH-responsive delivery of hydrophobic drugs. Carbohydr. Polym..

[29] Jeong J.-P., Kim K., Yoon I., Jang S., Jung S. (2025). Multifunctional biodegradable films of caffeic acid–grafted succinoglycan and polyvinyl alcohol with enhanced antioxidant, antibacterial, and UV-shielding properties. Int. J. Biol. Macromol..

[30] Sun K., Wang Z., Jiang J., Li Z., Ye S. (2025). Thiol-functionalized dual network polysaccharide microgels for synergistic Patulin adsorption and gut-liver axis protection via delivery and controlled release of Ginkgo Biloba extract. Chem. Eng. J..

[31] Chesneau C., Pelletier A., Dubot P., Leroy E., Goffin A., Jørgensen L., Hamadi S., Modjinou T., Houppe C., Wickramanayaka M.D. (2025). Synthesis and physico-chemical investigation of thiolated dextran derivative: Design and application of a redox-responsive drug delivery system. Int. J. Biol. Macromol..

[32] Pariguana M., Gonzalez L., de Loubens C., Vargas E., Marican A., Castro R., Cabrera-Barjas G., Durán-Lara E.F. (2025). Development of a Thiolated Carboxymethyl tara gum derivative with enhanced mucoadhesive and rheological behavior. Carbohydr. Polym. Technol. Appl..

[33] Wang L.-X., Wang Y., Pellock B., Walker G.C. (1999). Structural characterization of the symbiotically important low-molecular-weight succinoglycan of Sinorhizobium meliloti. J. Bacteriol..

[34] Xiao Y., Lu C., Liu Y., Kong L., Bai H., Mu H., Li Z., Geng H., Duan J. (2020). Encapsulation of Lactobacillus rhamnosus in hyaluronic acid-based hydrogel for pathogen-targeted delivery to ameliorate enteritis. ACS Appl. Mater. Interfaces.

[35] de Sousa I.P., Suchaoin W., Zupančič O., Leichner C., Bernkop-Schnürch A. (2016). Totally S-protected hyaluronic acid: Evaluation of stability and mucoadhesive properties as liquid dosage form. Carbohydr. Polym..

[36] Bahulkar S.S., Munot N.M., Surwase S.S. (2015). Synthesis, characterization of thiolated karaya gum and evaluation of effect of pH on its mucoadhesive and sustained release properties. Carbohydr. Polym..

[37] Lian H., Zhang T., Sun J., Pu X., Tang Y., Zhang Y., He Z. (2014). Enhanced paracellular and transcellular paclitaxel permeation by chitosan–vitamin E succinate–N-acetyl-l-cysteine copolymer on Caco-2 cell monolayer. J. Nanopart. Res..

[38] Yang N., Jike X., Zhang M., Jiang T., Lei H. (2025). Synthesis, characterization of thiolated hyaluronic acid and evaluation of its encapsulation effects on Limosilactobacillus reuteri HR7. Int. J. Biol. Macromol..

[39] Han X., Tian L., Chen Q., Wang L., Mi Y., Li Q., Guo Z., Dong F. (2025). Preparation of novel thiolated chitosan with significant antioxidant activity. Int. J. Biol. Macromol..

[40] Yalcintas E.P., Ackerman D.S., Korkmaz E., Telmer C.A., Jarvik J.W., Campbell P.G., Bruchez M.P., Ozdoganlar O.B. (2020). Analysis of In Vitro Cytotoxicity of Carbohydrate-Based Materials Used for Dissolvable Microneedle Arrays. Pharm. Res..

[41] Zhang F., Chen Y., Lü J., Liu R., Han H., Ma Y., Ma X., Yang J., Wang X., Lü X. (2024). Thiolated modified pectin for the efficient encapsulation of Companilactobacillus crustorum MN047. Food Hydrocoll..

[42] Santhanam S., Liang J., Baid R., Ravi N. (2015). Investigating thiol-modification on hyaluronan via carbodiimide chemistry using response surface methodology. J. Biomed. Mater. Res. Part A.

[43] Chen J., Ye F., Zhou Y., Zhao G. (2018). Thiolated citrus low-methoxyl pectin: Synthesis, characterization and rheological and oxidation-responsive gelling properties. Carbohydr. Polym..

[44] Hou L., Yu C., Zhang L., Zhang F., Linhardt R.J., Chen S., Ye X., Hou Z. (2023). Structure and microbial-modulating evaluation of a sulfhydryl-modified pectin. Food Hydrocoll..

[45] Bakhtiyari M., Moosavi-Nasab M., Askari H. (2015). Optimization of succinoglycan hydrocolloid production by Agrobacterium radiobacter grown in sugar beet molasses and investigation of its physicochemical characteristics. Food Hydrocoll..

[46] Moosavi-Nasab M., Taherian A.R., Bakhtiyari M., Farahnaky A., Askari H. (2012). Structural and rheological properties of succinoglycan biogums made from low-quality date syrup or sucrose using agrobacterium radiobacter inoculation. Food Bioprocess Technol..

[47] Staroszczyk H., Sztuka K., Wolska J., Wojtasz-Pająk A., Kołodziejska I. (2014). Interactions of fish gelatin and chitosan in uncrosslinked and crosslinked with EDC films: FT-IR study. Spectrochim. Acta Part A.

[48] Yu Y., Peng Y., Zhong Y., Su Z., Vijayakumar S., Chen Y., Mao Y., Wang L., Xin M., Li M. (2025). Innovative pH-responsive chitosan hydrogel: A systematic study on VB12 controlled release and multifunctional biological effects via synergistic grafting of cysteine and arginine. Int. J. Biol. Macromol..

[49] Chouly C., Colquhoun I.J., Jodelet A., York G., Walker G.C. (1995). NMR studies of succinoglycan repeating-unit octasaccharides from Rhizobium meliloti and Agrobacterium radiobacter. Int. J. Biol. Macromol..

[50] Carvalho I.C., Mansur A.A., Carvalho S.M., Florentino R.M., Mansur H.S. (2019). L-cysteine and poly-L-arginine grafted carboxymethyl cellulose/Ag-In-S quantum dot fluorescent nanohybrids for in vitro bioimaging of brain cancer cells. Int. J. Biol. Macromol..

[51] Zhang X., Ma J., Zheng B., Du Z., Zhang Y., Zhao G., Zhang L., Zang J. (2025). A disulfide-crosslinked pectin-glutenin hydrogel with advanced reversibility and toughness for cell culture. Int. J. Biol. Macromol..

[52] Gao H., Yang L., Tian J., Huang L., Huang D., Zhang W., Xie F., Niu Y., Jin M., Jia C. (2021). Characterization and rheological properties analysis of the succinoglycan produced by a high-yield mutant of Rhizobium radiobacter ATCC 19358. Int. J. Biol. Macromol..

[53] Nanaki S.G., Spyrou K., Veneti P., Karouta N., Gournis D., Baroud T.N., Barmpalexis P., Bikiaris D.N. (2022). L-cysteine modified chitosan nanoparticles and carbon-based nanostructures for the intranasal delivery of Galantamine. Polymers.

[54] Zhang Z., Mu L., Li J., Zhao H., Hou H.-M., Zhang G.-l., Hao H., Bi J. (2025). A double cross-linked film based on carboxymethyl chitosan binding with L-cysteine/oxidized konjac glucomannan with slow-release of nisin for food preservation. Food Chem..

[55] Kaur H., Yadav S., Ahuja M., Dilbaghi N. (2012). Synthesis, characterization and evaluation of thiolated tamarind seed polysaccharide as a mucoadhesive polymer. Carbohydr. Polym..

[56] Luo Q., Han Q., Wang Y., Zhang H., Fei Z., Wang Y. (2019). The thiolated chitosan: Synthesis, gelling and antibacterial capability. Int. J. Biol. Macromol..

[57] Grewal P., Mundlia J., Ahuja M. (2019). Thiol modified Moringa gum—A potential bioadhesive polymer. Carbohydr. Polym..

[58] Shen J., Li B., Zhan X., Wang L. (2018). A one pot method for preparing an antibacterial superabsorbent hydrogel with a Semi-IPN structure based on tara gum and polyquaternium-7. Polymers.

[59] Li X., Zhang X., Zhao W., Tian P., Tulugan K. (2024). Preparation of hirudin-loaded chitosan/polycaprolactone bowl-shaped particles and an application for a drug delivery system. Appl. Sci..

[60] Leitner V.M., Walker G.F., Bernkop-Schnürch A. (2003). Thiolated polymers: Evidence for the formation of disulphide bonds with mucus glycoproteins. Eur. J. Pharm. Biopharm..

[61] Yang J., Wang S. (2023). Polysaccharide-based multifunctional hydrogel bio-adhesives for wound healing: A review. Gels.

[62] Davoudi Z., Kali G., Braun D., Azizi M.H., Bernkop-Schnürch A. (2025). Highly thiolated corn starch for enhanced mucoadhesion and permeation. Int. J. Pharm..

[63] Bai L., Liu F., Xu X., Huan S., Gu J., McClements D.J. (2017). Impact of polysaccharide molecular characteristics on viscosity enhancement and depletion flocculation. J. Food Eng..

[64] Bamigbade G., Ali A.H., Subhash A., Tamiello-Rosa C., Al Qudsi F.R., Esposito G., Hamed F., Liu S.-Q., Gan R.-Y., Abu-Jdayil B. (2023). Structural characterization, biofunctionality, and environmental factors impacting rheological properties of exopolysaccharide produced by probiotic Lactococcus lactis C15. Sci. Rep..

[65] Meng Q., Wang Q., Chen L., Li J., Fan L., Gu Z., Shi G., Ding Z. (2023). Rheological properties and thickening effect of high molecular weight exopolysaccharide and intracellular polysaccharide from Schizophyllum commune. Food Hydrocoll..

[66] Kopač T., Ručigaj A., Krajnc M. (2022). Effect of polymer-polymer interactions on the flow behavior of some polysaccharide-based hydrogel blends. Carbohydr. Polym..

[67] Rana S., Upadhyay L.S.B. (2020). Microbial exopolysaccharides: Synthesis pathways, types and their commercial applications. Int. J. Biol. Macromol..

[68] Andhare P., Delattre C., Pierre G., Michaud P., Pathak H. (2017). Characterization and rheological behaviour analysis of the succinoglycan produced by Rhizobium radiobacter strain CAS from curd sample. Food Hydrocoll..

[69] Zhong L., Oostrom M., Truex M.J., Vermeul V.R., Szecsody J.E. (2013). Rheological behavior of xanthan gum solution related to shear thinning fluid delivery for subsurface remediation. J. Hazard. Mater..

[70] Wyatt N.B., Gunther C.M., Liberatore M.W. (2011). Increasing viscosity in entangled polyelectrolyte solutions by the addition of salt. Polymer.

[71] Ni Q., Ye W., Du M., Shan G., Song Y., Zheng Q. (2022). Effect of hydrogen bonding on dynamic rheological behavior of PVA aqueous solution. Gels.

[72] Bi H., Zhang X., Wang Q., Yong Q., Xu W., Xu M., Xu C., Wang X. (2023). Dynamic reversible disulfide bonds hydrogel of thiolated galactoglucomannan/cellulose nanofibril with self-healing property for protein release. Ind. Crops Prod..

[73] Sui Y., Jiang R., Niimi M., Hong J., Yan Q., Shi Z., Yao J. (2023). Development of dietary thiol antioxidant via reductive modification of whey protein and its application in the treatment of ischemic kidney injury. Antioxidants.

[74] Charlton N.C., Mastyugin M., Török B., Török M. (2023). Structural features of small molecule antioxidants and strategic modifications to improve potential bioactivity. Molecules.

[75] Ulrich K., Jakob U. (2019). The role of thiols in antioxidant systems. Free Radical Biol. Med..

[76] Knoll P., Le N.-M.N., Wibel R., Baus R.A., Kali G., Asim M.H., Bernkop-Schnürch A. (2021). Thiolated pectins: In vitro and ex vivo evaluation of three generations of thiomers. Acta Biomater..

[77] Rahmat D., Sakloetsakun D., Shahnaz G., Perera G., Kaindl R., Bernkop-Schnürch A. (2011). Design and synthesis of a novel cationic thiolated polymer. Int. J. Pharm..

